# The Effect of Laminectomy with Instrumented Fusion Carried into the Thoracic Spine on the Sagittal Imbalance in Patients with Multilevel Ossification of the Posterior Longitudinal Ligament

**DOI:** 10.1111/os.13147

**Published:** 2021-10-27

**Authors:** Kaiqiang Sun, Shikai Zhang, Benzhao Yang, Xiaofei Sun, Jiangang Shi

**Affiliations:** ^1^ Department of Spine Surgery Changzheng Hospital, Navy Medical University Shanghai Shanghai China; ^2^ Shanghai Kaiyuan Orthopedic Hospital Shanghai Shanghai China; ^3^ Department of Cardiology, Naval Medical Center Naval Medical University Shanghai China

**Keywords:** Cervical sagittal imbalance, Cervicothoracic junction, Laminectomy with instrumented fusion, Ossification of the posterior longitudinal ligament

## Abstract

**Objective:**

To determine if there is a difference in either the cervical alignment or the clinical outcomes in cervical ossification of the posterior longitudinal ligament (OPLL) patients who underwent laminectomy with instrumented fusion (LIF) ending at C_6_, C_7_, or proximal thoracic spine for the treatment of multilevel OPLL, and to find out the appropriate distal fusion level.

**Methods:**

This was a single‐center retrospective study. In total, 36 patients with cervical OPLL who underwent three or more level LIF in our institution between January 2015 and January 2017 were enrolled. They were divided into three groups according to their distal ends: C_6_ (nine females and 11 males, 60.45 ± 9.68 years old), C_7_ (four females and six males, 61.60 ± 10.29 years old), and T‐group (two females and four males, 64.33 ± 8.12 years old). Radiographic (compression level, classification of OPLL, occupying rate, C_2‐7_ cobb angle, C_2‐7_ sagittal vertical axis, and fusion level) and clinical outcomes (NDI score, operative time, and blood loss) were compared. Predictors of postoperative sagittal imbalance were also identified according to if the postoperative C_2‐7_ SVA was greater than 40 mm. The sensitivity and specificity of preoperative parameters predicting postoperative cervical stability were evaluated *via* the receiver operating characteristic (ROC) curve.

**Results:**

All patients were followed up at least 1 year. The blood loss in T group was significantly more than C_6_ or C_7_ group. The length of fusion level became significantly longer when the caudal level extended to the thoracic spine. The age, preoperative SVA, and NDI score at follow‐up were significantly greater in the imbalance group. At the final follow‐up, the cervical lordosis tended to be straight and the C_2‐7_ SVA tended to be greater when the caudal level of fusion was extended to upper thoracic segment. Further ROC curve analysis suggested that patients’ age had a sensitivity of 75.00%, specificity of 79.17% for cervical stability, and the AUC was 0.844 (*P* < 0.01), with the cutoff value for age being 66.5 years old. For preoperative SVA, the sensitivity was 58.30%, and specificity was 91.70%, with the AUC of 0.778 (*P* < 0.01). The cutoff value for preoperative SVA was 30.4 mm.

**Conclusion:**

Although posterior fusion terminating in the thoracic spine was not superior to the cervical spine for patients with multilevel OPLL, for elderly patients (>67 years) with great preoperative SVA (>30 mm), terminating at C_6_ was recommended to limit the invasion of cervical extensor muscles, provided the decompression was adequate.

## Graphical abstract

At the final follow‐up, the cervical lordosis tended to be straight and the C_2‐7_ SVA tended to be greater when the caudal level of fusion was extended to upper thoracic segment. However, for elderly patients (>67 years) with great preoperative SVA (>30 mm), terminating at C_6_ was recommended to limit the invasion of cervical extensor muscles.

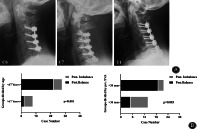

## Introduction

Cervical ossification of the posterior longitudinal ligament (OPLL) has ranked as one of the major contributors to progressive cervical myelopathy. Patients with cervical OPLL frequently exhibit such symptoms as weakness and/or numbness of the upper limbs, reduced manual dexterity, unstable gait, and bladder dysfunction, and also present with the following neurological signs: hyperreflexia, positive Hoffman's sign, positive plantar response, long tract signs, and lower limb spasticity^1^. Functional impairment in these patients can significantly reduce independence and quality of life. Among the various surgical procedures for cervical OPLL, laminectomy with instrumented fusion (LIF), has been widely used *via* indirect spinal cord decompression, especially for multi‐level (≥3 segments) OPLL^2^, ^3^, ^4^, ^5^. A great array of studies have reported that compared to cervical laminoplasty (CL), LIF could provide improvement and maintenance of cervical lordotic alignment and stronger biomechanical strength, which makes it more prevalent in clinical treatment of cervical myelopathy^2^, ^6^.

Notably, it is common that multi‐level cervical OPLL often extends to lower cervical segments, such as the C_6_ or C_7_ level. Thus, cervical fusion involving thoracic segment will be required. However, for patients with multilevel posterior cervical fusion, the most appropriate distal extent of fusion to improve sagittal alignment has always been a topic of debate among spine surgeons due to the anatomic and biomechanical differences between the cervical and thoracic spine. Anatomically, the cervicothoracic junction presents a unique biomechanical and structural characteristic, such as the lordotic cervical alignment changes to the kyphotic thoracic alignment and the abruptly sharp change in mobility of the spine^7^, ^8^. In addition, the cervical spine has a greater degree of mobility in flexion, extension, and side‐bending than the thoracic spine owing to articulation of the ribs and osteoligamentous structures in the thoracic spine^8^.

Although extensive research has been performed regarding the stabilization of the cervical spine, the optimal instruction for instrumentation at the cervicothoracic junction remains unknown. Long posterior cervical fusions may risk subjacent degeneration, spondylolisthesis, or kyphotic collapse when the fusion ends at the cervicothoracic junction^9^, ^10^. In cases with cervical spondylotic myelopathy, Schroeder and colleagues reported that the distal fusion level should be extended to T1 to decrease the revision rate^11^. However, another study suggested that constructs terminating in the proximal thoracic spine had similar revision rates and radiographic measurements as those terminating in the cervical spine^10^. The reasons regarding the destabilization in case of surgery involving the cervicothoracic junction can be concluded as follows: (i) decreased segmental motion *via* fusion may result in increased forces on adjacent segments^3^; (ii) posterior operations are more invasive to the posterior tension band, such as muscle dissection and laminectomies, which will further contribute to destabilization of the cervicothoracic junction^8^; and (iii) lack of the support from anterior column could limit the restoration of cervical lordosis *via* posterior approaches only^11^. Thus, recommendations for distal ending level of posterior cervical decompression and fusion remain variable and debated. Wha is more, to the best of our knowledge, there have been very few reports published to guide the appropriate distal fusion level in patients with multi‐level cervical OPLL.

Therefore, the purpose of this study is to determine: (i) if there is a difference in the cervical alignment in cervical OPLL patients who underwent LIF ending at C_6_, C_7_, or proximal thoracic spine for the treatment of multilevel OPLL; (ii) if there is a difference in the clinical outcomes in cervical OPLL patients who underwent LIF ending at C_6_, C_7_, or proximal thoracic spine for the treatment of multilevel OPLL; and (iii) to find out the appropriate distal fusion level.

## Methods

### Patients

The study was approved by the institutional review board (2014SL040), and all the patients have signed the informed consent. Patients with OPLL who underwent three or more level posterior LIF between January 2015 and January 2017 were included.

The inclusion criteria were as follows: (i) with the diagnosis of cervical OPLL based on patients’ clinical manifestations and concordant imaging examination; (ii) with complete medical records and follow‐up; (iii) treated by three or more level posterior LIF only; and (iv) without neurological symptoms resulting from spinal cord compression of thoracic or lumbar spine.

The following patients were excluded: (i) with congenital kyphosis, tumor, trauma, or potential infection; (ii) with the ossified mass extended to thoracic spine, (iii) without complete clinical data; and (iv) those followed up less than 1 year.

In total, 36 patients were enrolled in this study. According to the caudal level of fusion, the enrolled patients were divided into three groups: C_6_ group (fusion ending at C_6_ level), C_7_ group (fusion ending at C_7_ level) and T group (fusion ending into the thoracic spine).

### Evaluation of Radiological Parameters

All patients accepted imaging examination, including pre and last follow‐up X‐rays, CT and MRI.

#### Cervical Occupying Ratio

Preoperatively, the classification of OPLL and occupying radio (OR) of the spinal canal were measured on axial CT scans. Briefly, the occupying radio (OR) was defined as the thickness of OPLL divided by the anteroposterior diameter of the bony spinal canal at the maximum occupying level on an axial CT scan (OR = [the thickness of OPLL at the maximum occupying level/ the anteroposterior diameter of the bony spinal canal at the same level] × 100%)^12^. The larger the ratio, the more serious the compression will be.

#### Cervical Cobb Angle

Cervical alignment has become increasingly important in the planning of spine surgery, especially at cervicothoracic junction^13^. Therefore, the sagittal parameters were also assessed. Cervical lordosis, as the most frequently used parameter to evaluate the cervical alignment, was defined as the sagittal Cobb angle between the line drawn parallel to the inferior endplate of C_2_ and C_7_ vertebral bodies. The reported normal value of the Cobb angle was about 24 (range, 10° to 34°)^14^. Whether the Cobb angle is higher or lower than the normal value will reflect the bad cervical alignment.

#### C_2_ _‐7_ Sagittal Vertical Axis (SVA)

The C_2‐7_ sagittal vertical axis (SVA) is one of the most commonly used measures for cervical sagittal balance and is calculated by taking the horizontal distance between the postero‐superior corner of the C_7_ vertebral body and a plumb line drawn from the centroid of C_2_, which is correlated with quality‐of‐life parameters^15^.

All parameters were measured independently by three spine surgeons who were blinded to patient details. In fact, each spine surgeon was required to measure the radiological parameters for three times, and each time had a one‐week interval. If there was statistically significant difference among the three measurements for the same neurosurgeon, the parameter would be measured again. The average value of all three times was defined as the exact value of the parameter by the same neurosurgeon. Finally, the mean value of all the average values by the three spine surgeons was defined as the exact value of the parameter for analysis.

### Evaluation of Clinical Outcomes

Symptom duration and intra‐operative parameters (operative time, blood loss, and fusion level) were recorded.

Chronic cervical pain has been one of the most common postoperative symptoms after posterior surgery. In clinical practice and research setting, neck pain is often evaluated through patient reported measures, such as the Neck Disability Index (NDI). The NDI self‐report measure contains seven items related to activities of daily living, two items related to pain, and one item related to concentration (ability to read). Each item is scaled from 0 to 5, and the total score is expressed as a percentage, with higher scores representing greater levels of disability^16^. In general, NDI score can be categorized as: no disability (0%–8%), mild disability (10%–28%), moderate disability (30%–48%), severe disability (50%–68%), and complete disability (70%–100%)^17^.

The clinical outcome was measured by the neck disability index (NDI) preoperatively and at follow‐up.

### Statistical Methods

Statistical analysis was performed using SPSS21.0 (IBM, Armonk, NY, USA). The data were presented as the mean ± standard deviation. The least significant difference (LSD) test was used to compare the continuous variables among the C_6_, C_7_ and T groups. An independent‐sample *t* test was used to compare continuous variables between the balance and imbalance group. The Chi‐square test was used to compare the categorical variables. The sensitivity and specificity of preoperative parameters predicting postoperative cervical stability were evaluated *via* the receiver operating characteristic (ROC) curve. Values less than 0.05 (*P* < 0.05) were considered statistically significant.

## Results

### General Results

A total of 36 patients met the inclusion criteria with an average follow‐up of 1.3 years (range 1–2 years). 20 patients (nine females and 11 males) had multilevel posterior fusion terminating at C_6_ (average age: 60.45 ± 9.68 years; symptom duration: 12.95 ± 13.06 months; and follow‐up time: 1.28 ± 0.41 months); ten patients with the caudal level for C_7_ (average age: 61.60 ± 10.29 years; symptom duration: 14.20 ± 10.00 months; and follow‐up time: 1.30 ± 0.42 months), and six patients (two females and four males, average age: 64.33 ± 8.12 years; symptom duration: 12.33 ± 8.02 months; and follow‐up time: 1.42 ± 0.49 months) had the fusion terminating at the thoracic spine (Table 1).

**TABLE 1:** Demographic profile of patients in this study

	C_6_ group (*n* = 20)	C_7_ group (*n* = 10)	T group (*n* = 6)
Age	60.45 ± 9.68	61.60 ± 10.29	64.33 ± 8.12
Gender			
Female	9	4	2
Male	11	6	4
Symptom duration (month)	12.95 ± 13.06	14.20 ± 10.00	12.33 ± 8.02
Classification			
Segmental	4	3	2
Continuous	3	3	1
Mixed	13	4	3
Compression level	4.10 ± 1.02	4.00 ± 0.67	4.83 ± 1.17
OR (%)	52.80 ± 10.53	48.10 ± 13.73	47.56 ± 7.57
Preoperative C_2‐7_ Cobb	14.65 ± 11.58	10.11 ± 5.75	6.25 ± 3.84
Preoperative C_2‐7_ SVA (mm)	27.76 ± 11.82	27.75 ± 9.59	30.62 ± 7.17
Preoperative NDI score (%)	36.70 ± 4.51	36.40 ± 3.86	38.00 ± 5.22
Follow‐up time (year)	1.28 ± 0.41	1.30 ± 0.42	1.42 ± 0.49

NDI, neck disability index; OR, occupying ratio of the ossification of the posterior longitudinal ligament; SVA, sagittal vertical axis.

The radiological parameters and clinical outcome before operation were also shown in Table 1. The OR was 52.80% ± 10.53%, 48.10% ± 13.73%, and 47.56% ± 7.57% in the C_6_, C_7_, and T groups, respectively (*P* = 0.257). The preoperative C_2‐7_ Cobb was 14.65° ± 11.58°, 10.11° ± 5.75°, and 6.25° ± 3.84° in the C_6_, C_7_, and T groups, respectively (*P* = 0.335). The preoperative C_2‐7_ SVA was 27.76 ± 11.82 mm, 27.75 ± 9.59 mm, and 30.62 ± 7.17 mm in the C_6_, C_7_, and T groups, respectively (*P* = 0.439). In addition, The preoperative NDI score was 36.70% ± 4.51%, 36.40% ± 3.86%, and 38.00% ± 5.22% in the C_6_, C_7_, and T groups, respectively (*P* = 0.663) (Table 1).

No difference of the age, gender, symptom duration, follow‐up time, classification of OPLL, compression level, OR, preoperative C_2‐7_ Cobb, preoperative C_2‐7_ SVA, or preoperative NDI score was identified among the three groups.

#### Clinical Outcomes

Table 2 demonstrated the intraoperative parameters among the three groups. The operative time was 154.53 ± 78.39, 160.50 ± 32.87, 187.50 ± 20.43 min in the C_6_, C_7_, and T groups, respectively (*P* > 0.05). The blood loss in T group was significantly higher than C_6_ or C_7_ groups (641.67 ± 369.35 *vs* 370.00 ± 288.10, 641.67 ± 369.35 *vs* 305.00 ± 170.70) (*P* < 0.05).

**TABLE 2:** Comparison of intraoperative parameters among groups

	C_6_ group (*n* = 20)	C_7_ group (*n* = 10)	T group (*n* = 6)
Operative time (minutes)	154.53 ± 78.39	160.50 ± 32.87	187.50 ± 20.43
Blood Loss (mL)	370.00 ± 288.10	305.00 ± 170.70	641.67 ± 369.35*
Fusion level	4.05 ± 0.51**	5.10 ± 0.57**	6.83 ± 0.75**
C_2‐7_ Cobb at follow‐up	10.61 ± 12.13	11.05 ± 6.99	8.20 ± 4.80
C_2‐7_ SVA at follow‐up	35.98 ± 16.06	34.14 ± 10.74	38.42 ± 12.76
NDI score at follow‐up (%)	14.70 ± 4.91	16.60 ± 4.01	17.67 ± 1.51

NDI, indicates neck disability index; SVA, sagittal vertical axis.

* The blood loss in T group was significantly more than C_6_ or C_7_ group.

** The length of fusion level was significantly different among three groups.

### Radiological Outcomes

The length of fusion level was significantly different among the three groups, and it became longer when the caudal level extended to the thoracic spine (6.83 ± 0.75 *vs* 5.10 ± 0.57 *vs* 4.05 ± 0.51) (*P* < 0.05). With regard to the cervical alignment at the final follow‐up, the cervical lordosis tended to be straight and the C_2‐7_ SVA tended to be greater when the caudal level of fusion was extended to upper thoracic segment, but no significance was observed (Table 2).

### Representative Images

**Fig 1:**
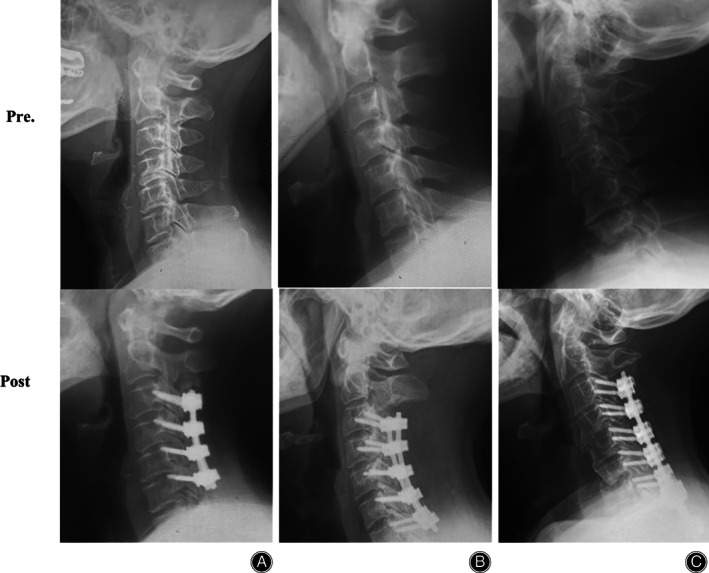
Representative images regarding the influence of different distal fusion level on radiographic outcomes. (A) Case 1, a 56‐year old male patient with continuous cervical OPLL from C_2_ to C_5_ was admitted to our institution. His preoperative NDI score was 36. Preoperative lateral X ray indicated that his cervical lordosis was 10.8° (A, upper). Laminectomy with instrumented fusion from C_3_ to C_6_ was given, with the distal fusion terminating at C_6_. At the final follow‐up, his NDI score improved to 14. And his cervical lordosis was 14.9°, with the change of Cobb angle of 4.1° (A, nether). (B) Case 2, a 53‐year old male patient with continuous cervical segmental OPLL from C_4_ to _C6_ was admitted to our institution. His preoperative NDI score was 37. Preoperative lateral X ray indicated that his cervical lordosis was 7.5° (B, upper). Laminectomy with instrumented fusion from C_3_ to C_6_ was given, with the distal fusion terminating at C_7_. At the final follow‐up, his NDI score improved to 16. And his cervical lordosis was 10.6°, with the change of Cobb angle of 3.1° (B, nether). (C) Case 3, a 46‐year old male patient with continuous cervical OPLL from C_3_ to C_7_ was admitted to our institution. His preoperative NDI score was 38. Preoperative lateral X ray indicated that his cervical lordosis was 11.9° (B, upper). Laminectomy with instrumented fusion from C_3_ to C_6_ was given, with the distal fusion terminating at T_1_. At the final follow‐up, his NDI score improved to 17. And his cervical lordosis was 10.4°, with the change of Cobb angle of −1.5° (B, nether).

Representative images were provided in Fig. 1 to show the influence of different distal fusion level on radiographic outcomes (Fig. 1). In addition, there was no statistical difference among the three groups in terms of NDI score at the final follow‐up.

### Analysis of Risk Factors for Postoperative Cervical Stability

#### Subgroup Analysis

Patients with cervical sagittal imbalance frequently complain of neck pain and functional disability, which predisposes surgery around the cervicothoracic junction to a unique set of challenges for the spine surgeon. Therefore, in this study, we further divided patients into postoperative cervical balance group and cervical imbalance group for the purpose of detecting the potential risk factors. The cervical sagittal balance was defined as the postoperative C_2‐7_ SVA larger than 40 mm^18^. Accordingly, the included cases were further divided into two groups: balance and imbalance group. As indicated in Table 3, no statistically significant difference was observed between the two groups except patients’ age and preoperative SVA. In the imbalance group, the average age was significantly higher than the balance group (67.67 ± 7.29 *vs* 58.29 ± 8.96), and the preoperative C_2‐7_ SVA was significantly greater (36.19 ± 8.34 *vs* 24.26 ± 9.03). Additionally, the NDI score at follow‐up was significantly greater in the imbalance group (18.17 ± 3.95 *vs* 14.50 ± 4.10).

**TABLE 3:** Comparison of parameters between balance and imbalance group

	Balance group (*n* = 24)	Imbalance group (*n* = 12)
Age	58.29 ± 8.96	67.67 ± 7.29*
Gender		
Female	11	3
Male	13	9
Symptom duration (month)	16.00 ± 12.75	10.25 ± 10.07
Classification		
Segmental	7	2
Continuous	3	4
Mixed	14	6
Compression level	4.29 ± 0.95	4.00 ± 1.04
OR (%)	50.02 ± 11.50	51.81 ± 10.61
Preoperative C_2‐7_ Cobb	12.07 ± 7.96	11.83 ± 12.91
Preoperative C_2‐7_ SVA (mm)	24.26 ± 9.03	36.19 ± 8.34*
Preoperative NDI scores (%)	36.17 ± 4.04	38.17 ± 4.86
Follow‐up time (year)	1.23 ± 0.36	1.46 ± 0.50
Fusion level	4.67 ± 1.17	5.08 ± 1.16
Distal fusion level		
C_6_	13	7
C_7_	8	2
T	3	3
Operative time (minutes)	165.67 ± 63.89	153.64 ± 56.92
Blood loss	395.83 ± 272.24	340.00 ± 340.45
C_2‐7_ Cobb at follow‐up	12.49 ± 9.34	6.01 ± 9.73
NDI score at follow‐up (%)	14.50 ± 4.10	18.17 ± 3.95*

* Statistically significant difference between groups.

NDI, neck disability index; OR, occupying ratio of the ossification of the posterior longitudinal ligament; SVA, sagittal vertical axis.

#### ROC Curve Analysis

**Fig 2:**
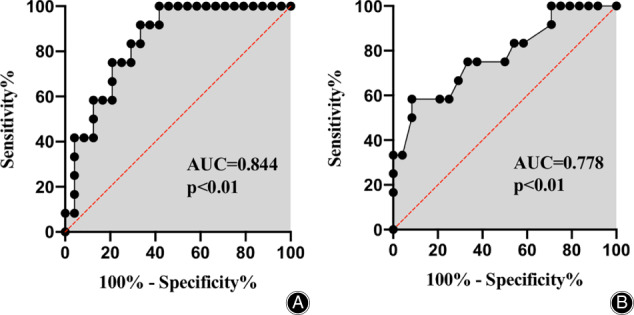
ROC curve regarding the effect of patients’ age and preoperative SVA on postoperative cervical stability. (A) The correlation of patients’ age with cervical stability (sensitivity:75.00%, specificity: 79.17%, and AUC: 0.844; *P* < 0.01; 95% confidence interval, 0.7182 to 0.9693). (B) The correlation of preoperative SVA with cervical stability (sensitivity: 58.30%, specificity: 91.70%, and AUC: 0.778; *P* < 0.01; 95% confidence interval, 0.6109 to 0.9446). AUC, area under curve; ROC, receiver operating characteristic; SVA, sagittal vertical axis.

**Fig 3:**
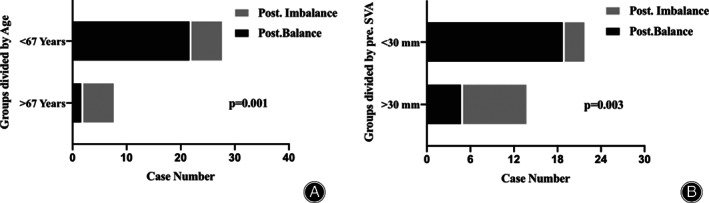
Case number of group divided by the cutoff value of age and SVA. (A) Case number of group divided by the cutoff value of age; (B) Case number of group divided by the cutoff value of SVA. SVA, sagittal vertical axis.

To validate the exact effect of patients’ age and preoperative SVA on postoperative cervical stability, we performed the ROC curve analysis, and the results suggested that patients’ age had a sensitivity of 75.00%, specificity of 79.17% for cervical stability, and the AUC was 0.844 (*P* < 0.01; 95% confidence interval (CI), 0.7182 to 0.9693). The cutoff value for age was 66.5 years old. For preoperative SVA, the sensitivity was 58.30%, and specificity was 91.70%, with the AUC of 0.778 (*P* < 0.01; 95% CI, 0.6109 to 0.9446) (Fig. 2). The cutoff value for preoperative SVA was 30.4 mm. Based on these cutoff values, we further divided the cases into high‐SVA (the preoperative C_2‐7_ SVA was greater than 30 mm) and low‐SVA group (the preoperative C_2‐7_ SVA was less than 30 mm), and age‐over 67 (the age was equal or greater than 67 years) and age‐below 67 group. The incidence rate of postoperative cervical sagittal imbalance was significantly greater in high‐SVA group and age‐over 70 (Fig. 3).

## Discussion

Laminoplasty has been one kind of widely used posterior approach for the treatment of multilevel and severe OPLL, but it may be related with delayed neurologic deterioration due to progressive kyphosis and progression of OPLL^3^. To provide stability of the decompressed levels, laminectomy with instrumented fusion (LIF) has been considered to be effective to restore cervical alignment and avoid kyphotic deformity^5^. However, when the fusion extended to the cervicothoracic junction, surgeons’ concern is raised due to its unique biomechanical and structural characteristic: should long‐segment cervical fusions be routinely carried out into the thoracic spine. However, to the best of our knowledge, there is a paucity of available literature on the question, especially for patients with cervical OPLL.

Choi and his colleagues recommended extending the distal fusion level to T_1_ to maintain the sagittal alignment, whereas they only analyzed the change of parameters before and after surgery, inter‐group differences have not been compared (Fusion to C_7_ *vs* Fusion to T_1_)^19^. Schroeder and colleagues also favored extending the multilevel cervical fusions to T_1_ ^11^. They concluded that patients whose construct terminated at C_7_ were 2.29 times more likely to require a revision than patients whose construct terminated at T_1_. However, in their study, cases that ended at C_6_ were excluded as those patients may bias the results: the revision rate was much lower for patients terminating at C_6_ than at C_7_. Interestingly, the C_2‐7_ SVA tended to be greater in the T_1_ group compared with C_7_ group in their study, though no significance has been identified, which was consistent with the present research. Contrary to the conclusion above, Truumees and his colleagues suggested that fusion end at both cervical and thoracic spine had the similar clinical and radiographic outcomes, while stopping in the cervical spine yielded length of hospital stay, estimated blood loss and operative time^10^. But they did not divide the cervical end level into C_6_ and C_7_. Kennamer and his colleagues’ study also showed that constructs terminating in the thoracic spine was not superior to those terminating in the cervical spine in terms of revision rates, NDI, and radiographic measurements^20^. In their research, patients were separated into four cohorts based on the caudal level of the fusion: C_6_ (or cranial), C_7_, T_1_, or T_2_ (or caudal). However, patients combined with anterior operation were not excluded. Thus, controversy exists in the few published studies. Moreover, all those articles focused on patients suffered degenerative cervical myelopathy, they obviously presented different biomechanical characteristic and prognosis compared with patients with multilevel OPLL. The results in this present study showed that stopping in the thoracic spine yielded high blood loss as results of longer fusion level, consistent with previous study^9^. Nevertheless, no significant difference was observed in terms of the NDI score and radiographic measurements at the final follow‐up among groups. Thus, we concluded that stopping in the thoracic spine was not superior to the cervical spine significantly in OPLL patients, and that it is not necessary to extend the fusion to the upper thoracic spine when the OPLL was limited in the cervical spine.

Cervical sagittal balance has been identified as an important determinant of radiological and clinical outcomes following cervical surgeries, which plays a critical role for keeping horizontal gaze and maintaining the whole spine balance^18^, ^21^. Furthermore, recent studies showed that postoperative cervical sagittal imbalance negatively affects outcomes of surgery in patients with cervical spondylotic myelopathy. Iyer and his colleagues’ study indicated that higher C_2‐7_ SVA was one of the independent predictors of worse preoperative NDI^22^. Tang and his colleagues proposed that C_2‐7_ SVA greater than 40 mm as cervical sagittal imbalance, and found statistically significant association between postoperative C_2‐7_ SVA and postoperative NDI and SF‐36 PCS scores^13^. Roguski and his colleagues’ study also showed that C_2‐7_ SVA were independent predictors of clinically significant improvement in SF‐36 PCS scores, and the majority of patients with C_2‐7_ SVA greater than 40mm did not improve from an overall health‐related quality of life perspective^23^. Accordingly, we defined the postoperative sagittal imbalance group as patients with the C_2‐7_ SVA greater than 40mm at the final follow‐up, and found that higher age and preoperative SVA correlated with postoperative sagittal imbalance which is consistent with previous reports^21^, ^23^, ^24^. Further ROC curve analysis suggested that patients aged more than 67 years or with higher preoperative SVA (>30 mm) tended to have higher risk of developing postoperative cervical stability. Surprisingly, caudal fusion involving the cervicothoracic junction did not lower the postoperative sagittal imbalance rate. In fact, the cervical extensor muscles play a critically important role in maintaining the cervical alignment. Researchers showed the high sagittal imbalance risky of the advanced age and the posterior approach surgery to the weakness and invasion of cervical extensor muscles^24^, ^25^. Therefore, we speculated that the same mechanism worked when the LIF was extended to the thoracic spine. A recent study also concluded that stopping at C_7_ did not negatively affect C_7_‐T_1_ segment failure, fusion rate, neck pain, neurologic outcomes, and cervical sagittal alignment. But this research demonstrated that postoperative NDI score was significant worse when fusion extending to the thoracic spine^26^. The contradiction may be attributed to the different follow‐up periods and grouping strategy. As shown in our study, the elderly patients (≥67) with great preoperative SVA (>30 mm) were vulnerable to the postoperative cervical sagittal imbalance. Therefore, based on the results of this study, terminating at C_6_ was recommended to limit the invasion of cervical extensor muscles, especially for patients whose age was more than 67 years old with preoperative SVA more than 30 mm, provided the decompression was adequate.

However, this study has the following limitations. First, it was retrospective in nature with a relatively small sample size and shorter duration of follow‐up. Due to the retrospective nature of this study, we could not give the exact indication for patients undergoing fusion ending at C_6_, C_7_ or thoracic spine. In fact, in our institution, the determination of the caudal level was frequently made by the surgeon's preference, as long as the stenotic levels were decompressed. As a result, in the section of the patients in our study, we did not include the corresponding description. However, we hope the results of our study could add some important information regarding the optimal distal fusion level. Second, we did not conduct a multivariate analysis to account for any confounders (such as patient comorbidities) in this study. In addition, in this study, we focused on patients with cervical OPLL, not all cervical myelopathy, such as severe cervical canal stenosis, which could also result in the relatively small sample size. Nonetheless, we controlled all our radiographic measurements as these data were measured by three independently experienced clinical spine surgeons *via* interval measurement. Despite the relatively small size, especially in the thoracic spine group, the results of this study also provide some important information for future high‐quality study. Third, the T_1_ slope can also be used to predict the sagittal alignment and neck disability effectively^27^. But this parameter was not chosen in the present study as T_1_ slope always cannot be measured as a result of the shielding of proximal thoracic at follow‐up. Finally, anterior fusion involving the cervicothoracic junction appeared to have lower sagittal imbalance and revision rate^28^. However, traditional anterior decompression cannot manage the multilevel OPLL well due to the technically difficulty and risky^24^, ^29^. Recently, a new technique named anterior controllable antedisplacement and fusion (ACAF) surgery was reported with satisfactory outcomes for patients with multilevel and severe OPLL^30^. This kind of anterior strategy was not included in this study. However, study investigating the clinical effect of ACAF with the caudal fusion level extended to upper thoracic spine on the recovery of cervical alignment and neurological function in cervical OPLL patients is being conducted.

### Conclusion

Posterior fusion terminating in the thoracic spine was not superior to the cervical spine significantly for patients with multilevel OPLL. Patients aged more than 67 years or with higher preoperative SVA (>30 mm) tended to have higher risk to develop postoperative cervical stability regardless of the caudal fusion level. Therefore, for those patients, terminating at C_6_ was recommended to limit the invasion of cervical extensor muscles, provided the decompression was adequate.
